# Supplementary material for: Applying time series analyses on continuous accelerometry data—A clinical example in older adults with and without cognitive impairment
Source: PLoS One. 2021 May 13;16(5):e0251544. doi: 10.1371/journal.pone.0251544 (PMC8118312; doi:10.1371/journal.pone.0251544)
Supplement: S1 File — (DOCX) [file pone.0251544.s001.docx]

**Circadian organization of activity patterns in mild cognitive impairment – a time series analysis**

Content

S1 [Table: Inclusion and exclusion criteria 2](#_Toc69069341)

S2 [Table: Neuropsychological test battery 3](#_Toc69069342)

[S1 Appendix: Statistical Appendix 3](#_Toc69069343)

S1 [Fig: Flow Chart 8](#_Toc69069344)

S2 [Fig: Positioning of actigraph device 9](#_Toc69069345)

S3 [Fig: Comparison of Daubechies wavelet and cubic B-splines basis functions 10](#_Toc69069346)

[References 12](#_Toc69069347)

## S1 Table: Inclusion and exclusion criteria

| **In- and exclusion criteria** | **MCI** | **Old** |
| --- | --- | --- |
| - 50 – 80 years of age | X | X |
| - Native German speakers | X | X |
| - No severe untreated medical, neurological or psychiatric diseases | X | X |
| - No contraindication to Magnetic Resonance Imaging | X | X |
| - No medication affecting central nervous system | X | X |
| - No recreational drug use | X |  |
| - daily consumption of > 50 g of alcohol or > 10 cigarettes | X | X |
| - No signs of subjective cognitive decline |  | X |
| - No signs of dementia (MMSE of ≥ 26 [1] and Consortium to Establish a Registry for Alzheimer’s Disease test battery (CERAD; Memory Clinic Basel, [www.memoryclinic.ch](http://www.memoryclinic.ch) [2]) < 1.5 standard deviation of age/education norms) |  | X |
| - Diagnosis of mild cognitive impairment § | X |  |
| - No Depression as monitored with Beck’s Depression Inventory II (exclusion if BDI-scores ≥ 13 [3]) |  | X |
| - No psychiatric comorbidities as monitored with State-Trait Anxiety Inventory (exclusion if STAI-X 1 score ≥ 40 [4]) |  | X |
| - No severe sleep disturbances | X | X |
| - No tumor or previous stroke as detected by acquired imaging | X | X |

§ MCI patients had to fulfill core clinical criteria for the diagnosis of MCI outlined by Petersen and others [5–7] which did not involve novel biomarkers as suggested in more recent MCI criteria [8]. Per criteria, MCI was required to report subjective and objective memory complaints, which were confirmed by standardized neuropsychological testing using CERAD and the Verbal Learning and Memory Test (VLMT [9]), but with preserved general function (MMSE>26), maintained independence and minimal if any impairment of function in daily life. Further, clinical assessment and structural MRI revealed no systemic or brain diseases accounting for declined cognition. Patients diagnosed with amnestic or amnestic plus MCI (in the following referred to as MCI) were included.

## S2 Table: Neuropsychological test battery

| Verbal memory |
| --- |
| - German version of the Auditory Verbal Learning Test (VLMT [9]) |
| Visual constructive memory |
| - Rey Osterrieth complex figure [10] |
| Working memory |
| - Wechsler Memory Scale [11] |
| Executive function |
| - Stroop color-word test [12] |
| - Trail Making Test Part B [13] |
| Attention |
| - Alters-Konzentrations-Test (AKT [14]) - Trail Making Test Part A [13] |

## S1 Appendix: Statistical Appendix

*Wavelet functions*

In wavelet analysis, the full set of wavelet basis functions is generated from a single wavelet *h(t)*, sometimes called the “mother” wavelet. Details are described in [15] pp. 56. In discrete wavelet analysis, time t changes in discrete steps. Since before further analysis the activity data were averaged over epochs of 5 minutes, t runs from t=0 to t=287 where t=0 corresponds to 0:00 (12 am), t=1 corresponds to 0:05 (12:05 am) and finally t=287 corresponds to 23:55 (11:55 pm). The Daubechies “mother” wavelet *h_t_=h(t)* of length 10 (d10), that generates all other wavelet basis functions, has only 10 contiguous non-vanishing values. For smoothing, we used the low-pass part $Y_{lp}$ of the wavelet transform matrix (the subscript *lp* stands for “**l**ow-**p**ass filter”). The columns of $Y_{lp}$ are the basis functions used in function-on-scalar regression. The column vectors of $Y_{lp}$ form a set of orthonormal basis functions. An activity record of a study participant for a 24h period is represented as a vector $x$ with 288 components. The smoothed data vector $x_{sm}$ of a 24 h activity record $\boldsymbol{x}$ is

$$x_{sm}=Y_{lp}Y_{lp}^{t}x$$

where $Y_{lp}^{t}$ denotes the transposed matrix of $Y_{lp}$ and juxtaposition of matrices means matrix multiplication.

*Function on scalar regression*

We can re-express model equation

$$x_{ij}\left( t \right)=b_{i}+\beta_{0}\left( t \right)+\sum_{k=1}^{p} \beta_{k}\left( t \right)\xi_{ik}+\varepsilon_{ij}\left( t \right); i=1,\ldots,N; j=1,\ldots,j_{i}; t=0,\ldots, 287. [1]$$

using the expansion of the coefficient functions $\beta_{k}$

$$\beta_{k}=\gamma_{k}Y_{lp}^{t} k=0,\ldots,p [2]$$

in matrix notation:

$X=b+\Xi\gamma Y_{lp}^{t}+\varepsilon$ [3]

*X*, *b*, *Ξ*, *γ* and *ε* are constructed from vectors and matrices in [1] by row stacking:

$X=\left( \begin{matrix} X_{1} \\ \vdots\\ X_{N} \end{matrix} \right)$ with

$X_{1}=\left( \begin{matrix} x_{11}\left( 0 \right) & \cdots& x_{11}\left( 287 \right) \\ \vdots& & \vdots\\ x_{1j_{1}}\left( 0 \right) & \cdots& x_{1j_{1}}\left( 287 \right) \end{matrix} \right)$ , …. , $X_{N}=\left( \begin{matrix} x_{N1}\left( 0 \right) & \cdots& x_{N1}\left( 287 \right) \\ \vdots& & \vdots\\ x_{Nj_{N}}\left( 0 \right) & \cdots& x_{Nj_{N}}\left( 287 \right) \end{matrix} \right)$.

and

$b=\left( \begin{matrix} B_{1} \\ \vdots\\ B_{N} \end{matrix} \right)$ with

$B_{1}=\left( \begin{matrix} b_{1} & \cdots& b_{1} \\ \vdots& & \vdots\\ b_{1} & \cdots& b_{1} \end{matrix} \right)\in\mathbb{R}^{j_{1}⨯288}$, … , $B_{N}=\left( \begin{matrix} b_{N} & \cdots& b_{N} \\ \vdots& & \vdots\\ b_{N} & \cdots& b_{N} \end{matrix} \right)\in\mathbb{R}^{j_{N}⨯288}$

$B_{1}$ is a constant matrix with $j_{1}$ rows and 288 columns and $B_{N}$ is a constant matrix with $j_{N}$ rows and 288 columns. Note, *j_i_* is the number of full 24 hours activity records case *i* contributed to the sample.

$\Xi=\left( \begin{matrix} \Xi_{1} \\ \vdots\\ \Xi_{N} \end{matrix} \right)$ with

$\Xi_{1}=\left( \begin{matrix} 1 & \xi_{11} & \cdots& \xi_{1p} \\ \vdots& \vdots& & \vdots\\ 1 & \xi_{11} & \cdots& \xi_{1p} \end{matrix} \right)\in\mathbb{R}^{j_{1}⨯(p+1)}$, … , $\Xi_{N}=\left( \begin{matrix} 1 & \xi_{N1} & \cdots& \xi_{Np} \\ \vdots& \vdots& & \vdots\\ 1 & \xi_{N1} & \cdots& \xi_{Np} \end{matrix} \right)\in\mathbb{R}^{j_{N}⨯(p+1)}$.

Note, $\Xi_{i}$ (*i*=1, … ,N) consists of $j_{i}$ identical rows that contain the values of the covariates of case *i*.

$$\gamma=\left( \begin{matrix} \gamma_{0} \\ \vdots\\ \gamma_{p} \end{matrix} \right)\in\mathbb{R}^{(p+1)⨯18}$$

$\varepsilon=\left( \begin{matrix} \varepsilon_{1} \\ \vdots\\ \varepsilon_{N} \end{matrix} \right)$ with

$\varepsilon_{1}=\left( \begin{matrix} \varepsilon_{11}\left( 0 \right) & \cdots& \varepsilon_{11}\left( 287 \right) \\ \vdots& & \vdots\\ \varepsilon_{1j_{1}}\left( 0 \right) & \cdots& \varepsilon_{1j_{1}}\left( 287 \right) \end{matrix} \right)$, … ,$\varepsilon_{N}=\left( \begin{matrix} \varepsilon_{N1}\left( 0 \right) & \cdots& \varepsilon_{N1}\left( 287 \right) \\ \vdots& & \vdots\\ \varepsilon_{Nj_{N}}\left( 0 \right) & \cdots& \varepsilon_{Nj_{N}}\left( 287 \right) \end{matrix} \right)$

The model equation [3] is not in standard form of a mixed model equation since on the left hand side is a matrix with $\sum_{i=1}^{N} j_{i}$ rows and 288 columns and not a column response vector. In order to convert [3] in the standard form of a mixed model equation, we must apply the vectorization operator **vec** to both sides. Vectorising a matrix *A* means stacking the columns of A into one column vector. The **vec**-operator is linear and has the following property ([16] Theorem 2, p. 35): Let A, B, C matrices for which the product ABC exists, then

$\mathrm{vec}\left( ABC \right)=\left( C^{t}\otimes A \right)\mathrm{vec}\left( B \right),$ [4]

where ⊗ denotes the Kronecker product (c.f. [16] Chapter 2, p. 31).

Applying this theorem to equation [3] leads to

$\mathrm{vec}\left( X \right)=\mathrm{vec}\left( b \right)+\left( Y_{lp}\otimes\Xi\right)\mathrm{vec}\left( \gamma\right)+\mathrm{vec}(\varepsilon)$ [5]

The estimation $\hat{\gamma}$ of the fixed effects parameters in $\mathrm{vec}\left( \gamma\right)$ can be carried out using the function **lme** from the **R** package **nlme**. Further,

$$Y_{lp}\hat{\gamma}^{t}=\left( \begin{matrix} \hat{\beta}_{0}(0) & \cdots& \hat{\beta}_{p}(0) \\ \vdots& & \vdots\\ \hat{\beta}_{0}(287) & \cdots& \hat{\beta}_{p}(287) \end{matrix} \right)=(\hat{\beta}_{0}^{t},\ldots, \hat{\beta}_{p}^{t})$$

is a matrix with 288 rows and p+1 columns where the columns are estimates of the coefficient functions in model equation [1]. The **R** function **vcov** returns the covariance matrix $\hat{V}$ of $\mathrm{vec}\left( \hat{\gamma} \right)$. Let $K={(k_{0},\ldots,k_{p})}^{t}$ be any real vector of length p+1 then

$\hat{\beta}=K^{t}\left( \begin{matrix} \hat{\beta_{0}} \\ \vdots\\ \hat{\beta_{p}} \end{matrix} \right)$=$K^{t}\hat{\gamma}Y_{lp}^{t}$ [6]

is an arbitrary linear combination of the coefficient functions. Applying again [4] gives

$\hat{\beta}=\mathrm{vec} \left( \hat{\beta} \right) =\mathrm{vec} \left( K^{t}\hat{\gamma}Y_{lp}^{t} \right)=\left( Y_{lp}\otimes K^{t} \right)\mathrm{vec} \left( \hat{\gamma} \right)$ [7]

From [7] follows that the covariance matrix of $\hat{\beta}$ is

$Cov( \hat{\beta})$= ($Y_{lp}\otimes K^{t})\hat{V}{(Y_{lp}\otimes K^{t})}^{t}$ [8].

Most applications require only the variances $Var( \hat{\beta})=\left( \mathrm{Var} \left( \hat{\beta}\left( 0 \right) \right),\ldots,\mathrm{Var} \left( \hat{\beta}\left( 287 \right) \right) \right)^{t}$, which are the diagonal elements of the 288x288 matrix $Cov( \hat{\beta}):$

$Var( \hat{\beta})=\mathrm{diag} \left( (Y_{lp}\otimes K^{t})\hat{V}{(Y_{lp}\otimes K^{t})}^{t} \right)$ [9]

The operator **diag** extracts the vector of diagonal elements of a square matrix.

Confidence bands and p-values

From equations [6] and [9] of the appendix follow the formula for (1-α)100% confidence bands (intervals) for β.

$\begin{aligned} \mathrm{CI}\left( 1-\alpha\right)100\%=\left( \hat{\beta}-z_{1-\frac{\alpha}{2}}\sqrt{Var( \hat{\beta})}; \hat{\beta}+z_{1-\frac{\alpha}{2}}\sqrt{Var( \hat{\beta})} \right) \\ \end{aligned}$ [10]

where $z_{1-\alpha/2}$ is the 1-α/2 quantile of the standard normal distribution. $\hat{\beta}$ and $Var( \hat{\beta})$ were calculated using formula [6] and [9], respectively.

For each i=0 ,…, 287 the null hypothesis

$H_{0}:\beta\left( i \right)=0, i=0,\ldots,287$ [11]

is tested by a Z-Test. β is an arbitrary linear combination of the coefficient functions β_0_,…, β_p_ with coefficients in K. The Z-test statistic is

$Z_{i}$=$\frac{\hat{\beta}(i)}{\sqrt{\mathrm{Var} \left( \hat{\beta}(i) \right)}}$ = $\frac{{{(K}^{t}\hat{\gamma}Y_{lp}^{t})}_{i}}{\sqrt{{\mathrm{diag} \left( (Y_{lp}\otimes K^{t})\hat{V}{(Y_{lp}\otimes K^{t})}^{t} \right)}_{i}}}$ *i=*0,..,287 [12]

and the two-sided p-value of the Z-Test is

$P_{i}=2\Phi\left( -\left| Z_{i} \right| \right)$ [13]

where $\Phi$ is the standard normal probability distribution.

## S1 Fig: Flow Chart


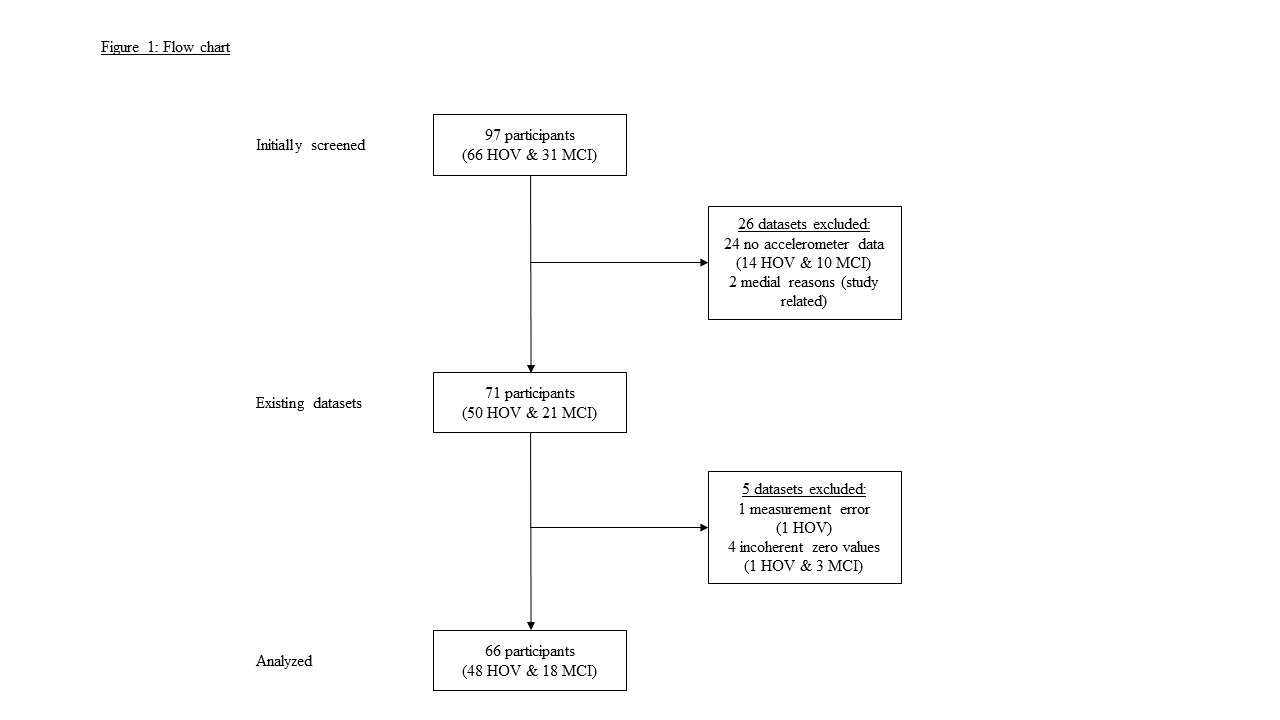


## S2 Fig: Positioning of actigraph device


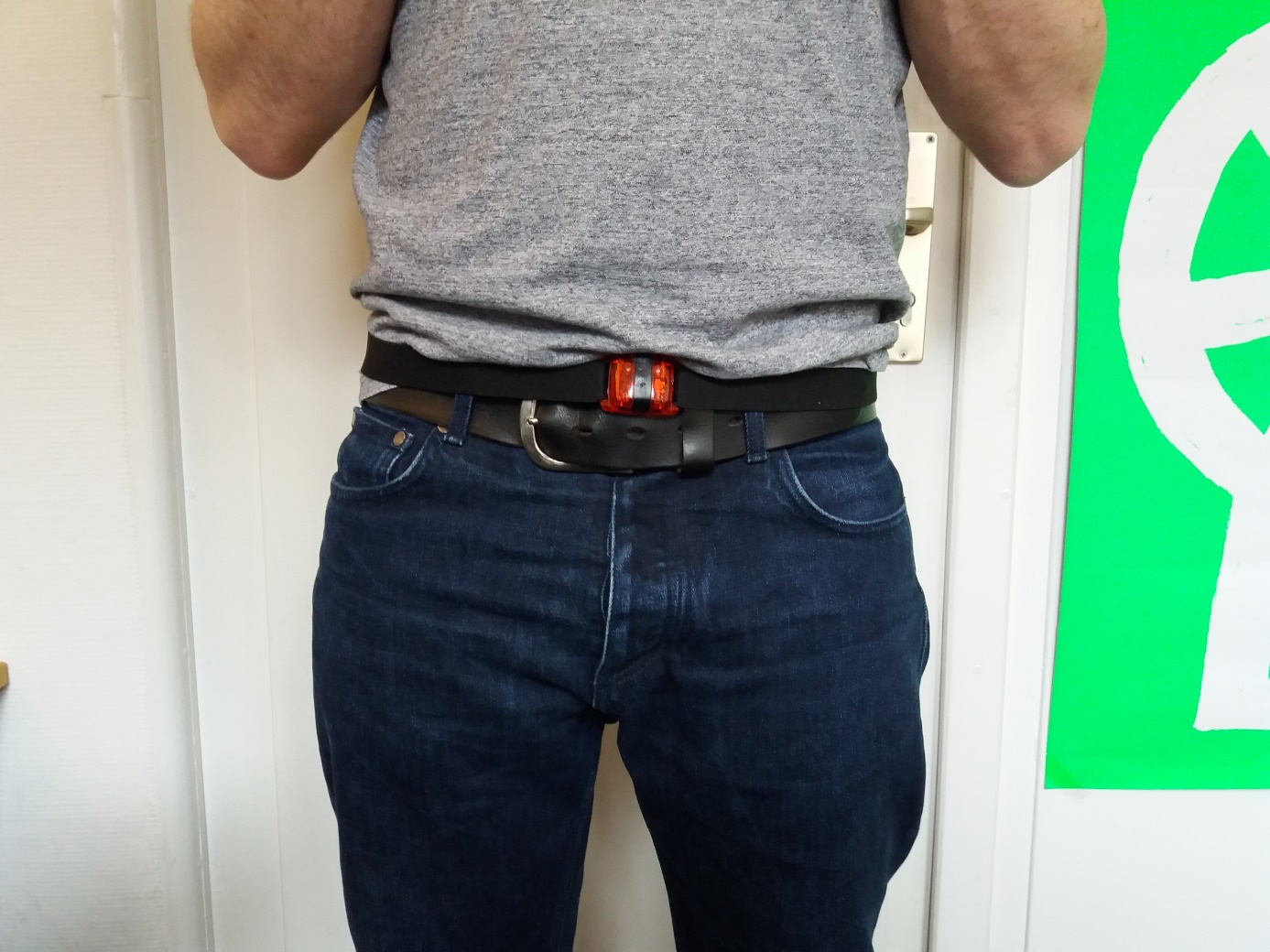


## S3 Fig: Comparison of Daubechies wavelet and cubic B-splines basis functions

**A)**


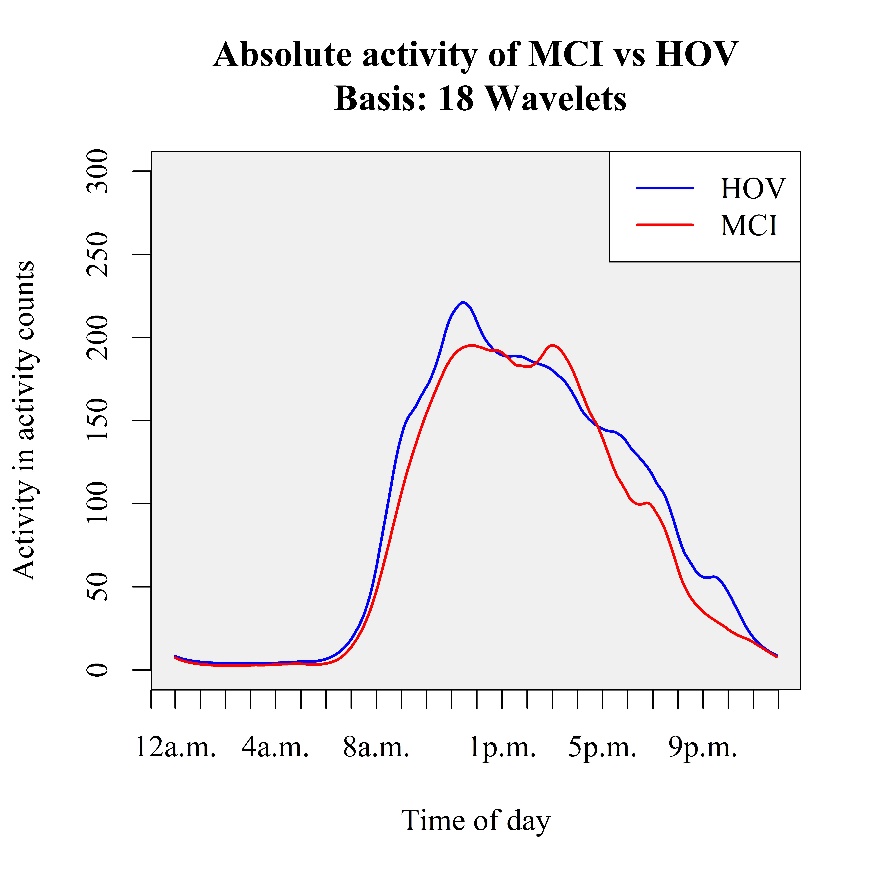


S3A Fig: Time course of absolute activity in activity counts between MCI and HOV. FoSR is modelled with 18 Daubechies wavelet basis functions.

**B)**


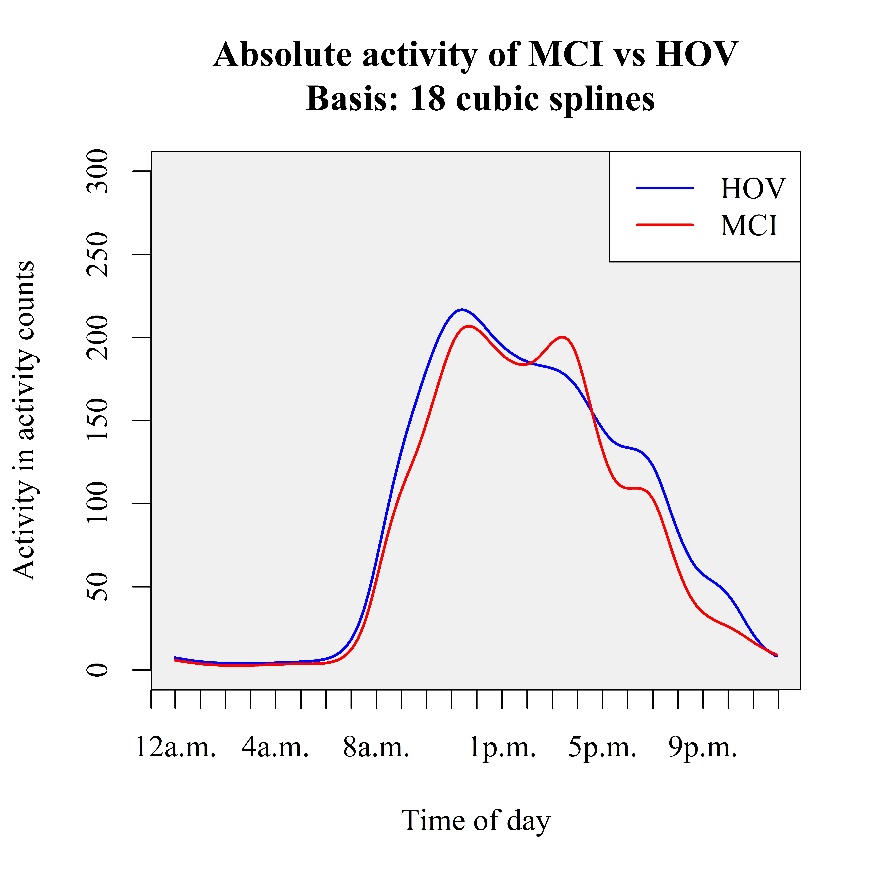


S3B Fig: Time course of absolute activity in activity counts between MCI and HOV. FoSR is modelled with 18 cubic B-spline basis functions based on the refund package.

# References

1. Folstein MF, Folstein SE, McHugh PR. “Mini-mental state”. A practical method for grading the cognitive state of patients for the clinician. J Psychiatr Res. 1975;12(3):189–98.

2. Morris JC, Heyman A, Mohs RC, Hughes J, van Belle G, Fillenbaum G, et al. The Consortium to Establish a Registry for Alzheimer ’ s Disease Neuropathology confirmation of the. Neurology. 1989;39(9):1159–65.

3. Kühner C, Bürger C, Keller F, Hautzinger M. Reliabilität und Validität des revidierten Beck-Depressionsinventars (BDI-II). Nervenarzt. 2007 Jun 7;78(6):651–6.

4. Spielberger CD, Gonzales HP, Taylor CJ, Anton WD, Agaze B, GR R. Test Anxiety Inventory: Preleminary professional manual. Palo Alto, CA: Consulting Psychologists Press. 1980.

5. Petersen RC. Mild cognitive impairment as a clinical entity and treatment target. Arch Neurol. 2004;62(7):1160–3; discussion 1167.

6. Winblad B, Palmer K, Kivipelto M, Jelic V, Fratiglioni L, Wahlund L-O, et al. Mild cognitive impairment--beyond controversies, towards a consensus: report of the International Working Group on Mild Cognitive Impairment. J Intern Med. 2004;256(3):240–6.

7. Petersen RC, Doody R, Kurz A, Mohs RC, Morris JC, Rabins P V, et al. Current concepts in mild cognitive impairment. Arch Neurol. 2001 Dec;58(12):1985–92.

8. Albert MS. Changes in cognition. Neurobiol Aging. 2011;32(SUPPL. 1):S58–63.

9. Helmstaedter C, Lendt M, Lux S. Verbaler Lern- und Merkfähigkeitstest. Göttingen (Germany): Beltz; 2001.

10. Stern R, Singer E, Duke L, Singer N, Morey C, Daughtrey E. The Boston qualitative scoring system for the Rey-Osterrieth complex figure: Description and interrater reliability. Clin Neuropsychol. 1994;8:309–22.

11. Wechsler D. Wechsler Adult Intelligence Scale - Third Edition. San Antonio (TX): Psychological Corporation; 1997.

12. Van der Elst W, Van Boxtel MPJ, Van Breukelen GJP, Jolles J. The Stroop color-word test: influence of age, sex, and education; and normative data for a large sample across the adult age range. Assessment. 2006;13(1):62–79.

13. Tombaugh TN. Trail Making Test A and B: Normative data stratified by age and education. Arch Clin Neuropsychol. 2004;19(2):203–14.

14. Gatterer G. Alters-Konzentrations-Test (AKT) (2nd restandardized edition). Göttingen (Germany): Hofgrefe; 2008.

15. Percival DB, Walden AT. Wavelet Methods for Time Series Analysis. Cambridge: Cambridge University Press; 2000.

16. Magnus JR, Neudecker H. Matrix differential calculus with applications in statistics and econometrics. John Wiley; 1999. 395 p.
