# Supplementary material for: Applying time series analyses on continuous accelerometry data—A clinical example in older adults with and without cognitive impairment
Source: PLoS One. 2021 May 13;16(5):e0251544. doi: 10.1371/journal.pone.0251544 (PMC8118312; doi:10.1371/journal.pone.0251544)
Supplement: Attachment — Submitted filename: PONE-D-20-08099.docx [file pone.0251544.s002.docx]

PLOS ONE

Applying time series analysis on continuous accelerometry data-a clinical example in older adults with and without cognitive impairment

This paper examines activity levels in a small sample of older adults with the goal of identifying differences in activity patterns between those with normal cognition and those with Mild Cognitive Impairment. Activity data come from accelerometers worn on the hip for up to seven days. The paper argues that the methods used—function on scalar regression—allow them to characterize activity patterns. These patterns are compared for older adults with normal cognition to those of older adults with MCI. The comparisons are then redone within categories of “morning”, “intermediate” and “evening” chronotypes—that is those who characterize themselves a more active and alert in the morning, between morning and evening, and evening.

The strengths of the paper include the detailed accelerometry data on a clinical sample and the application of methods that seem to be well suited to characterizing activity patterns. The weaknesses of the paper include the very small sample size (47 patients with normal cognition and 13 with MCI), the extreme selectivity in the analysis sample compared to the clinical sample, and the failure to adjust for or to characterize this selectivity, especially for the MCI patients, less than half of whom survived deletion for various reasons. I was worried in particular about the loss of MCI patients because of “incoherent zero values.” Perhaps these are a result of cognitive limitations, which is exactly what you are trying to study. The sample was reduced also by “insufficient wear time”, which could also be due to cognitive issues. These problems with selectivity of the sample, with no attempt to characterize it or correct it, make the results suspect.

The paper also is motivated by assessing an approach that “might be able to detect subtle changes” but the data are not change data. They are differences in the cross section. It also points to early detection as valuable to allow treatments, but no treatments for cognitive decline currently exist. The Introduction promises hypotheses about differences in activity patterns for older adults with normal cognition vs those with MCI but presents none. It does not discuss the mechanisms through which cognitive limitation might affect activity patterns.

I also worried about dividing a sample of 13 patients with MCI by chronotype and then comparing this tiny group to any other group. It looks like fishing for significance.

I would suggest a paper that keeps all the patients in the original sample. Why drop those with MRIs that suggest a stroke or other organic cause of the cognitive problems? Do you NOT expect those people to show activity changes? Dial back the claims of the analysis. Say you are looking for differences in the cross-section that might be worth (eventually, in another study) more investigation. Produce some hypotheses (or maybe one). Test it. Forget the chronotype. That’s for later. Unless you have strong theory to support differences in the relationship between MCI and activity for morning people vs others. And I certainly didn’t see it in the paper. Finally, drop all the descriptives on the variables you don’t use, like personality. These are not of interest here.
