# Supplementary material for: Applying time series analyses on continuous accelerometry data—A clinical example in older adults with and without cognitive impairment
Source: PLoS One. 2021 May 13;16(5):e0251544. doi: 10.1371/journal.pone.0251544 (PMC8118312; doi:10.1371/journal.pone.0251544)
Supplement: Attachment — Submitted filename: Response to reviewers.docx [file pone.0251544.s004.docx]

Dear Dr. Oyster,

Thank you very much for giving us the opportunity to revise our manuscript. We are confident that we are able to fulfill PLOS One requirements with this revised version.

Of note, the new requests from reviewer #1 deal with

-	questioning the epoch length we chose of 5 minutes

-	questioning to use wavelet functions for smoothing instead of cubic spline functions

-	questioning the number of used wavelet functions

The reviewer is often referencing the article by Goldsmith et al. and the refund r package and is asking for the rationale and evidence for our decisions to use wavelet functions instead of cubic splines, an epoch length of 5 minutes instead of 10 minutes as in the article by Goldsmith and colleagues and evidence for the number of wavelet functions we used. We understand that these are relevant and crucial points and we tried our best to clarify and justify our decisions. However, we want to point out that also Goldsmith et al. are not providing more rationale and evidence for the epoch length or the kind of smoothing functions compared to us, and that our analysis and an analysis based on the refund package with similar calculations as used by Goldsmith et al. (see below) yielded very similar results. Insofar, we think we answered the questions of the reviewer in the best way possible.

Please find below our responses and respective changes to our revised manuscript.

Best,

Torsten Rackoll

Reviewer #1:

The purpose of this paper is to use Function on Scalar Regression to determine if activity levels between healthy older volunteers and individuals with Mild Cognitive Impairment throughout different times of the day.

I believe the authors have done a good job of addressing my initial comments. However, with further inspection of the cited research on FOSR and code provided new concerns are presented.

Comment #1:

In my second comment I asked the reviewers to provide a rationale for why they use a 5-minute epoch which they have now provided. Looking at the original Goldsmith paper (11) their rationale for epoch length is not based on activity patterns but rather achieving a normal distribution of count data at each time point. This comment by Goldsmith represents an important statistical consideration and the authors should confirm the same is true for their sample given the smaller sample size used here compared to Goldsmith (11). Unintended outliers that are generated at certain timepoints due to a selected epoch length may skew results of the FOSR analysis. You state that you log transform the data to correct for skew but that may be an unnecessary step if the right epoch is chosen. Also, you state in response to comment 1 that your focus is on longer phases of activity over the day, yet you choose a shorter epoch than previous studies see Goldsmith (11).

We thank Reviewer #1 for allowing us to further clarify how our approach (rationale given in the previous version) relates to Goldsmith et al.

First, we concede that pre-investigating the distribution of the data is important to minimize risks of bias due to outliers. However, an approach that chooses epoch lengths with the aim to achieve normally distributed data might lead to impractical epoch length (e.g. seven minutes could be the smallest epoch length with normally distributed data but a 24 hour cycle cannot be divided into seven minute epochs). Thus, our decision on epoch length was not driven by the aim to achieve a normal distribution of count data at each time point. We decided to define an epoch length on conceptual grounds in which we aimed “to detect differences in activity patterns over the course of a day” (for details see our response from the last revision) and to subsequently use data transformations that avoid skewness. Log-transformation is a simple data transforming step that is easy to apply, while the chances of introducing bias are limited, rendering it an appropriate and economic strategy for other clinical researchers (Bland & Altman, BMJ, 1996). Of note, also Xiao and other authors from the ‘refund’ package log-transformed their count data due to skewness (Xiao et al, Biostatistics, 2015).

Second, our comment from the previous revision (“focusing on identifying longer periods”) was related to the alternative of using very short epochs such as one minute. We revised the wording for our rationale in the Methods section from our previous revision by deleting the word ‘longer’. The respective sentence now reads as follows:

*“We summarized the activity data using 5 minutes epochs resulting in 288 count data sets per participant per day (24h=1440 minutes=288*5 minutes)(10). The rationale for the 5 minutes epoch was that we aimed to identify group differences of activity levels over the course of a day, focusing on identifying periods with high/middle/low physical activity, respectively.”*

Comment #2:
In response to comment 3 you state your design of your wavelet function is based on your hypothesis that MCI patients would have higher activity at night and lower during the morning compared to HOV. This is not your hypothesis in the manuscript, however. On line 110 you make a more general hypothesis that your algorithm will detect differences in activity in both timing and magnitude between the two groups. No where in the introduction do you state any evidence on how circadian rhythms would be disrupted in terms of specific time of day. If there is evidence it needs to be stated. Your hypothesis would also need to be updated in the manuscript. It is wearisome that you use one hypothesis to justify a methodological decision in the comments but then state a different hypothesis in the manuscript.

We want to use the opportunity to clarify a misunderstanding from comments given in our last revision. In our comment #3 from our last response, we compared 40 minute intervals (36 basis functions) with 80 minute (18 basis functions) and 120 minute intervals (9 basis function). Our choice of the number of basis functions was based on our hypothesis that we would thus detect group difference that are evenly distributed over the course of a day. We considered an underlying time window that was neither too small nor too large. In our statement in comment #3 we wanted to give an example of what time distributions we were aiming at. Within that sentence we accidently left out the word ‘for example’. It should have stated: “Given our hypothesis that the circadian rhythm of MCI patients would be disturbed, with, for example, higher activity during night and lower activity in the morning hours compared to healthy older controls, we wanted to examine activity for longer time-intervals than one hour only.” Previous evidence indicates that differences in activity patterns are differentially spread over the day given circadian disruptions observed in patients with Alzheimer’s disease (e.g., increased wake after sleep onset and delayed wake times, Leng et al*. Lancet Neurol*, 2020; Musiek et al. *Jama Neurol*, 2018). There is not enough evidence on rest-activity cycles in MCI patients and also no strong indication how that influences activity patterns. Therefore, we would like to stay with the hypothesis provided in the manuscript and apologize for given a misleading explanation in the last revision. Data are not sufficient to ground a hypothesis on specific periods in which activity differs between MCI and HOV.

Comment #3:
More to the point, your rationale for using a wavelet function is based on research that uses this type of function on time series data but not necessarily research that has used FOSR. Goldsmith (11) provides all the code necessary to use FOSR as it was applied in their paper which seems to be the fundamental goal of this paper, i.e. use FOSR to determine differences in activity patterns between MCI and HOV volunteers. However, the Goldsmith R package, refund, does not include a function to use wavelets for smoothing data, which is why I believe you go this route of using a linear mixed effects model instead. Ultimately, this smoothing step is of huge importance in the application of FOSR where the level of smoothing may produce differences between groups that do not necessarily exist. You justify the use of the wavelet function based on previous research using it on time series data but has it ever been used on a geriatric population? Or have the number of basis functions been validated to represent true activity versus noise? Obviously, a wavelet function with X number of basis functions may be more accurate for one group or individual than another, for example, 36 basis functions for kids versus 9 for older adults. If you have no direct evidence validating the application of wavelets with a specific number of basis functions to the activity levels of the groups investigated here, I would recommend dropping this smoothing step altogether. Alternatively, I recommend that the authors utilize the Goldsmith package, refund, to implement FOSR as it has been used in the past, thus making the methods of this paper more directly comparable to the research they claim to base the current results off of. Additionally, the use of the refund package would allow for more direct model comparison between models that include or exclude the covariate of group while controlling for age and sex. Code to do all this can be found here: <https://jeffgoldsmith.com/papers.html>. CRAN for refund here: <https://cran.r-project.org/web/packages/refund/refund.pdf>.

The reviewer addresses a valid point with respect to methodological aspects of the statistical approach. First, we want to highlight that the aim of our manuscript is to introduce clinical researchers to the advantages of FoSR for the assessment of accelerometer-based time series activity data. Although further research is needed on different aspects of the FoSR model statistics, validating the approach is not the goal of our current study and might obscure the manuscript’s agenda for the clinical reader. Reviewer #1 suggested to use the refund package as done by Goldsmith et al., as the choice of basis functions is not based on validation studies, and might therefore not be the optimal choice of model selection. We followed this suggestion and performed the respective analyses. Results were very similar. Please find a comparison between the FoSR with Daubechies Wavelets as basis functions and cubic b-splines as basis functions in figure 1 and figure 2, respectively. Per default, the refund package performs FoSR with cubic b-splines and 15 basis functions. Fifteen basis functions are similar to the choice of 18 basis functions made in our approach. Of note, the authors of the refund package do not ground their choice of basis functions or their numbers on statistical considerations.

Figure 1: Time course of absolute activity in activity counts between MCI and HOV. FoSR is modelled with 18 Daubechies wavelet basis functions.

Figure 2: Time course of absolute activity in activity counts between MCI and HOV. FoSR is modelled with 18 cubic B-spline basis functions based on the refund package.

Although we do acknowledge that to some extent more research could improve Daubechies Wavelets in use of FoSR, we believe that several critical points raised by Reviewer #1 are equally true for cubic splines. E.g., the choice of basis functions used is to some extent arbitrary. Additionally, it is assumed that activity patterns follow locally a polynomial progression of degree 3. Daubechies wavelets are 24 h periodic discrete functions and thus more suitable to model activity data that follows a 24h rhythm. The coefficient functions from FoSR analysis with cubic splines are 24 h periodic only because non-periodicity is averaged out in large samples.

We agree that the comparison FoSR with wavelets versus FoSR with cubic splines might be of interest for the informed reader so we added respective comparison in the supplementary appendix.

Comment #4:

The number of basis functions used in your wavelet transformation also dictates your capability to determine statistical significance at different times based on your use of a bonferonni correction to account for multiple comparisons. Why not use a false discovery rate correction instead? Applying Bonferroni based on number of basis functions seems to make it either too conservative or too liberal and has a direct impact on your capability to determine when the activity patterns differ between groups. Also, the refund package uses an FOSR function that is Bayesian and therefore utilizes confidence intervals to make determinations of significance rather than p-values.

The choice of correction for multiple testing is indeed an area in which more research might enhance the informative value of the FoSR. Of note, currently no such correction is implemented in the refund package. We here improved the model from Goldsmith et al to some extent. Bayesian methods also do not solve the problems arising from high dimensionality and bounding the FDR instead of the FWER does not change the fact that any method to adjust 288 highly correlated p-values tends to be too conservative. We compared our FoSR with correction for multiple comparison after Bonferroni-Holm (FWER) to one with Benjamini-Yekutieli (FDR). Both correction methods displayed the same time window with statistically significant differences in activity but FDR extended the time frame by ten minutes. Both methods are rather conservative but led to similar results. To spread awareness for the need to further validate different model choice in FoSR modelling we now added a sentence to the Discussion which now reads as follows:

*Forth, model choices in FoSR such as number of basis functions or correction methods for multiple testing should be validated in different patient populations to enhance the informative value of the regression method.*

Comment #5:
The authors also never run a multiple linear regression on the aggregate activity data. In the introduction the authors state that this method is in effectual but they never perform it to actually demonstrate that this is the case.

A comparison with well-known multiple regression assists the reader in putting the new approach into perspective. We thank reviewer #1 for this suggestion. The reason why we did not present this analysis in our manuscript was that a multiple linear regression on aggregated activity data does not take into account differential activity pattern over the time course of the day which we believe to be important. Therefore, we think that this comparison is actually misleading as it might suggest that the FoSR is a better regression model than a multiple linear regression. But the comparison is not correct in the first place as the linear model treats activity data as an absolute number and the FoSR treats it as a function of time-based activity values. We performed a linear mixed regression adjusted for age and sex on the average activity counts per day as a dependent variable and group as a covariate. The respective linear regression model was now added to the Methods and the Results section which now read as follows:

*We performed a multiple linear regression with average activity count as a dependent variable and group as an explanatory variable adjusted for age and sex.*

*Based on the simple linear regression model, average activity count was similar in both groups (-7 counts for MCI compared to HOV, 95% CI -20 to 7, p-value = 0.32).*

Comment #6:
Change hours to hour, line 110.

We did the requested change.

Comment #7:
Be sure to cite the NHANES cohort, line 174.

We cited the NHANES 2003-2004 cohort accordingly:

Centers for Disease Control and Prevention National Center for Health Statistics. NHANES Questionnaires, Datasets, and Related Documentation [Internet]. [accessed 2021 Feb 20]. Available from: https://wwwn.cdc.gov/nchs/nhanes/continuousnhanes/default.aspx?BeginYear=2003

Comment #8:
Please cite who is suggesting that the use of cubic splines or wavelet transformations is suggested for actigraph data, line 206.

We added the requested references:

Xiao L, Zipunnikov V, Ruppert D, Crainiceanu C. Fast covariance estimation for high-dimensional functional data. Stat Comput. 2016;26(1–2):409–21.

Leise TL. Analysis of Nonstationary Time Series for Biological Rhythms Research. J Biol Rhythms. 2017;32(3):187–94.
